# Comparing standard versus enhanced implementation of an evidence-based HIV prevention program among Bahamian sixth grade students: findings from nationwide implementation trials

**DOI:** 10.1186/s12889-022-13848-9

**Published:** 2022-07-29

**Authors:** Bo Wang, Lynette Deveaux, Carly Herbert, Xiaoming Li, Lesley Cottrell, Richard Adderley, Maxwell Poitier, Arvis Mortimer, Glenda Rolle, Sharon Marshall, Nikkiah Forbes, Bonita Stanton

**Affiliations:** 1 Department of Population and Quantitative Health Sciences, University of Massachusetts Chan Medical School, 368 Plantation Street, Worcester, MA 01605 USA; 2 Office of HIV/AIDS, Ministry of Health, Shirley Street, Nassau, Bahamas; 3 Department of Health Promotion, Education, and Behavior, University of South Carolina Arnold School of Public Health, 915 Greene Street, Suite 408, Columbia, SC 29208 USA; 4 Department of Pediatrics, West Virginia University, 959 Hartman Run Road., Morgantown, WV 26506 USA; 5 Department of Pediatrics, Wayne State University School of Medicine, 400 Mack Avenue, Detroit, MI 48201 USA; 6 Hackensack Meridian School of Medicine, 340 Kingsland, St., Nutley, NJ 07110 USA

**Keywords:** Enhanced implementation, Implementation strategies, Evidenced-based intervention, Implementation fidelity, HIV prevention, The Bahamas

## Abstract

**Background:**

Effective implementation strategies are needed to address the challenges encountered by teachers in implementation of evidence-based HV prevention programs in schools. The current study: 1) compares implementation fidelity of *Focus on Youth in the Caribbean (FOYC)* plus *Caribbean Informed Parents and Children Together (CImPACT)* intervention using enhanced implementation strategies (including biweekly monitoring/feedback and site-based mentoring) to those using more traditional approach (teacher training only); and 2) evaluates the impact of school coordinators’ and mentors’ performance on teachers’ implementation fidelity and student outcomes.

**Methods:**

Data from an enhanced implementation trial in 2019–2020, involving 24 government primary schools, 79 teachers, and 2252 students, were compared to data from a standard implementation trial in 2011–2012, involving 35 government primary schools, 110 teachers and 2811 students using mixed-effects modeling and structural equation modeling.

**Findings:**

Teachers in the 2019–2020 trial taught more core activities (28.3 vs. 16.3, t = 10.80, *P* < 0.001) and sessions (7.2 vs. 4.4, t = 9.14, *P* < 0.001) than those participating in the 2011–2012 trial. Teachers taught > 80% of the intervention curriculum in 2019–2020 compared to 50% curriculum delivery in 2011–2012. Teachers who had a “very good” or “excellent” school coordinator in their schools taught more core activities than those who had a “satisfactory” school coordinator (30.4 vs. 29.6 vs. 22.3, F = 18.54, *P* < 0.001). Teachers who worked in a school which had a “very good” mentor, taught more core activities than those teachers who did not have a mentor or had only a “satisfactory” mentor (30.4 vs. 27.6; t = 2.96; *p* = 0.004). Teachers’ confidence in implementing core activities, comfort level with the curriculum, attitudes towards sex education in schools, and perceived principal support were significantly related to increased self-efficacy, which in turn was related to teachers’ implementation fidelity. The degree of implementation was significantly associated with improved student outcomes.

**Implications/conclusion:**

An evidence-based HIV prevention intervention can achieve a high degree of implementation when delivered with enhanced implementation strategies and implementation monitoring. Future program implementers should consider the purposeful selection and training of school coordinators and mentors to support low-implementing teachers as a potentially important strategy when attempting to achieve high-quality implementation of school-based interventions.

## Contributions to the literature

1. Our study investigated the implementation of an evidence-based intervention by comparing data from standard and enhanced implementation trials, which allows assessment of the relative contribution of an enhanced implementation approach to both implementation fidelity and program outcomes.
2. Our study demonstrated that an evidence-based HIV prevention intervention can achieve a high degree of implementation when delivered with enhanced implementation strategies and implementation monitoring.
3. Purposeful selection and training of school coordinators and mentors to support low-implementing teachers is a potentially important strategy when attempting to achieve high-quality implementation of school-based interventions.
4. These findings address recognized gaps in knowledge regarding effective implementation strategies and teacher implementation support (biweekly monitoring and feedback, coaching/peer mentoring).

## Introduction

### Challenges in the implementation of school-based prevention programs

The school setting offers great opportunities for the implementation of health promotion programs, including HIV prevention and sex education. Numerous evidence-based school prevention programs have been developed and evaluated over the last several decades, showing significant impacts on students’ knowledge, attitudes, and health behaviors [1, 2]. While many school-based programs have shown great success in improving student health-related behavior in randomized controlled trials, there have been significant challenges in implementing these programs in real-life settings outside of the trial environment, the so-called “implementation gap” [3, 4]. School-based programs are especially prone to adaptations in the real-world setting, as compared to programs implemented in research and healthcare settings [5]. Adaptations can improve intervention fit to the setting while maintaining effectiveness [6]. However, heavy modifications (> 1/4 core activities) were found to be negatively related to teachers’ implementation fidelity and diminished program effectiveness [7]. Low quality of intervention delivery can be attributed to many factors, including academic pressures, time constraints, and competing priorities at the teacher, school, and district levels [5, 8]. Implementation strategies specific to the school setting are needed to address these unique challenges and barriers.

### Relationship between implementation Fidelity and student outcomes

Over the past decade, implementation science researchers have sought to address this implementation gap, utilizing implementation fidelity as a measurement system for standardization [9]. Implementation fidelity is defined as the degree to which an intervention is implemented as intended [10]. High implementation fidelity has been positively correlated with student outcomes [11–13]. Conversely, as implementation fidelity decreases, many programs demonstrate diminishing results [14]. Implementation fidelity has been measured across five dimensions: 1) adherence, 2) dose, 3) quality of program delivery, 4) participant responsiveness, and 5) program differentiation (inclusion of distinguishing factors of the program) [10]. Among a random sample of 342 teachers in the United States implementing a substance use prevention program, almost no teachers demonstrated implementation fidelity on all five dimensions, with only 25% of teachers demonstrating adherence to the program and 33% implementing the curriculum on the recommended schedule [15]. Teachers demonstrated even lower levels of fidelity in the other domains of quality, participant responsiveness, and program differentiation [15]. Further, an evaluation of the implementation of a sexual and reproductive health intervention in 649 schools in Tanzania revealed that nearly 70% of schools failed to implement about 50% of the intended course materials [16]. Lack of implementation fidelity can weaken program outcomes or result in diminished program effectiveness [17] . Thus, it is important to understand the factors that impede and enhance high quality implementation of evidence-based interventions in schools.

### School- and teacher-level factors associated with teachers’ implementation

Principal support, compatibility with scheduling demands, mission-policy alignment, teacher training, and school culture are important in determining success of implementation on the school level [3, 4, 17, 18]. Teachers cite time restrictions and scheduling challenges as major reasons for deviation from fidelity [19]. In an educational system with competing demands, “such deviation” often reflects teachers’ prioritization of certain educational topics [3]. Teachers have also reported decreasing fidelity as implementation complexity increases [20]. Teachers’ individual traits, including motivation, enthusiasm, comfort with the topics addressed, and self-efficacy, also play a large role in implementation fidelity [8, 19, 21, 22]. Vanwesenbeeck et al. (2016) found that teachers who were uncomfortable with the material but responsible for implementing a comprehensive sex education program would frequently skip or shorten lessons on more sensitive topics [8].

### Effective implementation strategies to enhance teachers’ content delivery

Teacher training and provision of technical assistance have been shown to be particularly effective strategies in improving implementation fidelity and addressing implementation challenges in the school setting. Amount and quality of teacher training impacts both program content (e.g., core activities) and number of sessions taught [23, 24]. Technical assistance, including strategies such as coaching, web-based support, performance feedback, and peer support, has been successfully utilized to improve implementation fidelity and support teachers during the intervention implementation process [18, 25–27]. In a school-based sex education and pregnancy prevention study, the use of a comprehensive technical assistance program (i.e., Fidelity through Informed Technical Assistance and Training [FITT]) resulted in an overall 98% of the curriculum delivery [28]. Coaching has also been shown to be an effective strategy in improving implementation fidelity and sustainability of school-based interventions [18, 29]. Positive relationships have been noted between amount of performance feedback and teacher’s quality of program delivery [18]. Teacher peer learning groups have been an effective tool during the implementation process, as they provide an opportunity for teachers to reflect, share, and debrief their experiences and challenges in meaningful ways [30]. Teacher peer learning groups have also been shown to help teachers learn and implement new, engaging teaching methods, which is necessary in the implementation of many school-based interventions [31].

### Implementation of focus on youth in the Caribbean (“FOYC”) and Caribbean informed parents and children together” (CImPACT) in the Bahamas

Focus on Youth (FOY) with Informed Parents and Children Together (ImPACT) was selected by the Prevention Research Synthesis project at the United States’ Centers for Disease Control and Prevention (CDC) as a Best Evidence Program and is included in the CDC’s “Diffusion of Behavioral Interventions” portfolio [32]. The US-Bahamian team developed and evaluated the Bahamian adaptation of FOY and ImPACT, an 8-session plus two booster sessions-adolescent HIV prevention intervention entitled “Focus on Youth in the Caribbean” (FOYC) and a 1-hour parental monitoring intervention entitled “Caribbean Informed Parents and Children Together” (CImPACT) [33]. Short- and long-term evaluations showed that the intervention significantly increased youth’s HIV/AIDS knowledge and condom use skills, as well as perceptions, intentions, and practices relevant to HIV prevention among Bahamian adolescents [34, 35]. Based on the effectiveness of the intervention through 36 months, the Bahamian Ministry of Education (MOE) decided to implement FOYC + CImPACT in all grade six classes in the government elementary schools throughout the nation, with annual follow-up booster sessions to be delivered in grades 7 through 9 in the government junior high schools. FOYC is delivered as part of the Health and Family Life Education (HFLE) curriculum, and CImPACT is incorporated into parent-teacher meetings [36].

Our research team conducted a national implementation study in 80 government elementary schools and 34 government middle (junior high) schools in The Bahamas from 2011 to 2016. We found that 208 grade-six teachers in 80 schools taught an average of 15.6 out of 30 core activities and 4.6 out of 8 sessions of FOYC [37], consistent with other school-based implementation of prevention programs [16]. Students taught by low-implementation teachers demonstrated poorer outcomes in regard to HIV/AIDS knowledge, reproductive health skills, self-efficacy, and behavioral intentions [37]. With support from the NIH, our team collaborated with The Bahamas Ministry of Education (MOE) to develop several culturally appropriate implementation strategies (e.g., biweekly monitoring and feedback, site-based mentorship and assistance) to increase teachers’ implementation fidelity and sustainability [38]. The MOE has deployed these innovative teacher training and support programs for the subsequent national implementation of FOYC+CImPACT in 66 government elementary schools (including the first wave of implementation in 24 schools in New Providence) since 2019. We employ an adapted version of Aaron’s and colleagues’ Exploration, Preparation, Implementation, Sustainment (EPIS) model to guide our implementation study as it is logical and evidence-based [39]. The EPIS model articulates variables that may play crucial roles at different phases in the implementation process. The model examines both the “outer context” (e.g., leadership, environment/policy, and funding) and the “inner context” factors (e.g., fidelity monitoring and support, providers’ characteristics and attitudes) with regard to the intervention. EPIS emphasizes the significant role of sustained leadership, ownership of new interventions, and ongoing support as key to successful implementation and sustainability.

#### Research questions

National implementation of FOYC+CImPACT in The Bahamas offers a unique opportunity to evaluate several implementation strategies and program outcomes. Drawing on data gathered through the first wave of national implementation in both trials, this analysis addresses three research questions: (1) Did teachers achieve increased implementation fidelity of the FOYC+CImPACT intervention with newly developed implementation strategies in 2019–2020, as compared to the standard implementation in 2011–2012? (2) How did variations in school coordinators and mentors’ performance and teacher-level factors influence the teacher’s implementation fidelity? and (3) How did implementation fidelity impact students’ outcomes?

## Methods

### Study site

In the Fall of 2019, all 24 elementary schools located on New Providence, the most populated islands in The Bahamas, participated in the first wave of national implementation. The 24 participating schools housed 79 grade six classes and teachers and 24 school coordinators. Vast majority (> 95%) of the teachers and school coordinators were female and African American. Approximately 22% of the teachers had 6–10 years of teaching experience while 63% had worked as teacher for 10–20 years. Approximately half of the 79 grade six teachers in 2019–2020 had participated in 2011–2012 implementation. The research protocol was approved by the University of Massachusetts Medical School (UMMS) Institutional Review Board and the Institutional Review Board of the Bahamian Princess Margaret Hospital, Public Hospitals Authority.

#### Teacher training

Sixty-one (77.2%) grade six teachers who teach Health and Family Life Education (HFLE) classes in New Providence schools completed a two-day teacher training workshop. The training was provided by three Bahamian Focus on Youth trainers who have extensive experience implementing FOYC+CImPCT and a US training specialist with expertise preparing educators to lead FOYC+CImPCT. The training focused on increasing the teachers’ curriculum knowledge, building positive attitudes about the curriculum, and increasing skills and comfort to deliver the curriculum. Consistent with the FOY training guidelines, the training was comprised of clear expressed objectives, short lecturettes, group discussions, videos from the curriculum, skill and curriculum demonstration, active learning through skill practice, role plays and teach backs [40]. The teacher training covered: 1) review of the need for HIV prevention in The Bahamas; 2) overview of FOYC including the research showing its effectiveness; 3) a “walk-through” of each session of FOYC with modeling of the “core” activities considered to be critical to the success of FOYC; 4) a didactic question-and-answer period regarding menstruation, contraception and condom-use; and, 5) a modeling of CImPACT, followed by implementation guidance. All teachers were given a copy of the FOYC teacher training manual and a FOYC+ CImPACT 24/7 flash drive for “point-of-care” guidance as they prepared the lessons.

### School coordinator and mentor training

Twenty-four school coordinators were identified and trained for the purpose of tracking teachers’ implementation and progress biweekly, collecting teacher’s measures, and identifying and reporting issues/problems to the researchers located in New Providence (called “biweekly monitoring and feedback”). Twelve high-performing teachers (mentors) were trained for the purpose of identifying the challenges faced by at-risk and moderate-performing teachers, assisting teachers in preparing for intervention sessions, and providing guidance to improve curriculum delivery (called “site-based assistance and mentorship”). The school coordinator and mentor trainings were conducted by two Bahamian trainers who have extensive experience implementing FOYC+CImPACT in October 2019. Both training sessions lasted 2 to 3 hours.

### Measures

#### Implementation fidelity

To assess implementation fidelity all teachers were asked to complete a Teacher Implementation Checklist specific for each of the eight FOYC sessions and CImPACT parent session after they had taught the session. Fidelity of implementation is defined as adherence to the core activities in this analysis because core activities that were identified by the developers are essential elements in FOYC+CImPACT that impact student outcomes [37]. The checklist includes core 35 activities. The teachers documented the activities that they taught in each session, and for the ones taught, their degree of comfort in teaching the lesson (very comfortable, somewhat comfortable, and not comfortable at all), whether they had modified the format of the activity outlined in the manual (lengthen, shorten and/or substantially change the activity), and how many students (most, some, and few) appeared to be engaged in the lesson.

#### Teacher’s characteristics, training experience, and perceptions

A pre-implementation questionnaire was used to collect information described in the extant research as influencing fidelity of intervention implementation: teacher’s level of formal education; years as a teacher; teacher’s attendance at FOYC training workshop; teachers’ perceptions of the importance of HIV prevention (very meaningful, somewhat meaningful, or not at all meaningful) for grade six students in their schools; teacher’s comfort level in teaching the FOYC+CImPACT intervention; and, whether teachers had had other competing lessons or teaching priorities. Performance of school coordinators and mentors at each school was assessed by New Providence school coordinator, Bahamas FOYC project managers and MOE education officer using brief questionnaire survey.

The pre-implementation questionnaire assessed teachers’ perceived *principal supportiveness* (four items) [41, 42], teachers’ *confidence* teaching/discussing five topics such as condom use, teen pregnancy and HIV/AIDS (five items) [43], teachers’ *attitudes towards sex education* in schools (eight items) [44], and teachers’ *self-efficacy* in teaching the FOYC+CImPACT intervention (three items) [45]. Answers are given on a Likert scale with five options (1 = totally disagree to 5 = totally agree). These scales have been validated with grade six teachers in Bahamian public schools in our pilot study. The internal consistency (Cronbach’s α) of the scales is adequate (principal supportiveness α = 0.85; confidence, α = 0.91; attitudes towards sex education, α = 0.68; self-efficacy α = 0.79).

#### Student outcomes

An anonymous curricular assessment instrument, adapted by the Ministry of Education (MOE) from a version of the Bahamian Youth Health Risk Behavioral Inventory (BYHRBI) [46], was administered to grade six students at the beginning of grade six before receipt of FOYC and at the beginning of grade 7 (the follow up was originally scheduled at the end of grade six but was delayed due to the COVID-19 pandemic). The instrument assessed HIV/AIDS knowledge and preventive reproductive health skills, as well as some perceptions, intentions, and self-reported behaviors. A 16-item scale including true and false statements was used to assess level of HIV/AIDS knowledge. Correct responses were scored 1 and incorrect 0, resulting in a summary score of 0 to 16 for each participant. Preventive reproductive health skills were assessed through an adaptation of the Condom-use Skills Checklist (e.g., “when people use a condom, the condom is unrolled before it goes on”) [47]. The validated scale includes true and false statements describing the steps of correct condom use from opening a condom pack for use to disposal after use. Correct responses were scored 1 and incorrect 0, resulting in a summary score of 0 to 6 for each participant. A five-item self–efficacy scale assessed youth’s beliefs about their own ability to use pregnancy/STI prevention methods (e.g., “I could get condoms”). Each statement has a 5-point response option ranging from “1 = strongly disagree” to “4 = strongly agree.” The internal consistency of the scale was 0.78. A composite score was calculated as a mean score across the five items (range 1 to 5). Intention to use condom protection was measured using the question, “what are the chances that you would use a condom if you need to prevent yourself from getting HIV?” Youth rated the likelihood on a 5-point Likert scale ranging from 1 (no chance in the world) through 5 (yes, big chance that I would).

### Analysis

Frequency distribution of numbers of sessions taught and number of core activities completed in the enhanced implementation trial in 2019–2020 and in the standard implementation trial in 2011–2012 was calculated. Histograms were then constructed to graphically display teachers’ implementation in standard versus enhanced implementation of the FOYC+CImPACT intervention. To identify factors that are associated with teachers’ implementation fidelity, we conducted a bivariate analysis (ANOVA and Student t test) to compare the number of core activities (from among a total of 35 possible) and the number of sessions taught (from among a total of nine possible) by teachers according to teacher characteristics, training and teaching experience, and performance of school coordinators and mentors. Pearson correlation analysis was conducted to examine the associations between implementation fidelity and factors influencing teacher’s implementation fidelity. The responses to anonymous student questionnaires were aggregated at classroom level and linked to the teachers’ data.

Linear mixed-effects model was used to examine the combined effects of teachers’ education, comfort level with the curriculum, confidence in implementing core activities, attitude towards sex education, perceived principal support, self-efficacy and performance of school coordinators and mentors on implementation fidelity, controlling for clustering effect of school. Bivariate analysis and mixed-effect modeling were performed to examine the association between teacher’s implementation and student outcome. Student’s t test was first used to analyze changes in student scores of AIDS knowledge, condom use skill, self-efficacy and intention from baseline to follow-up at the end of the school year. We calculated average scores of student outcomes for each classroom and examine the implementation dose and fidelity and student outcome using mixed-effects models.

Structural equation modeling (SEM) analysis was conducted to examine the relationships among factors influencing teacher’s implementation fidelity, and student outcomes using the Mplus 8. Standardized regression coefficients for all paths were estimated using robust maximum likelihood (MLR) estimation. Missing data was handled using full information maximum likelihood (FIML). Goodness of model fit was evaluated using chi-square to degrees-of-freedom ratio (χ^2^/df), root mean square error of approximation (RMSEA), Bentler’s comparative fit index (CFI) and Tucker Lewis Index (TLI) [48]. Acceptable model fit is determined by an RMSEA less than 0.08, and values of CFI and TLI greater than 0.90 [48].

## Results

### Comparison of teachers’ implementation fidelity between 2011 and 2012 trial and 2019–2020 trial

**Fig. 1:**
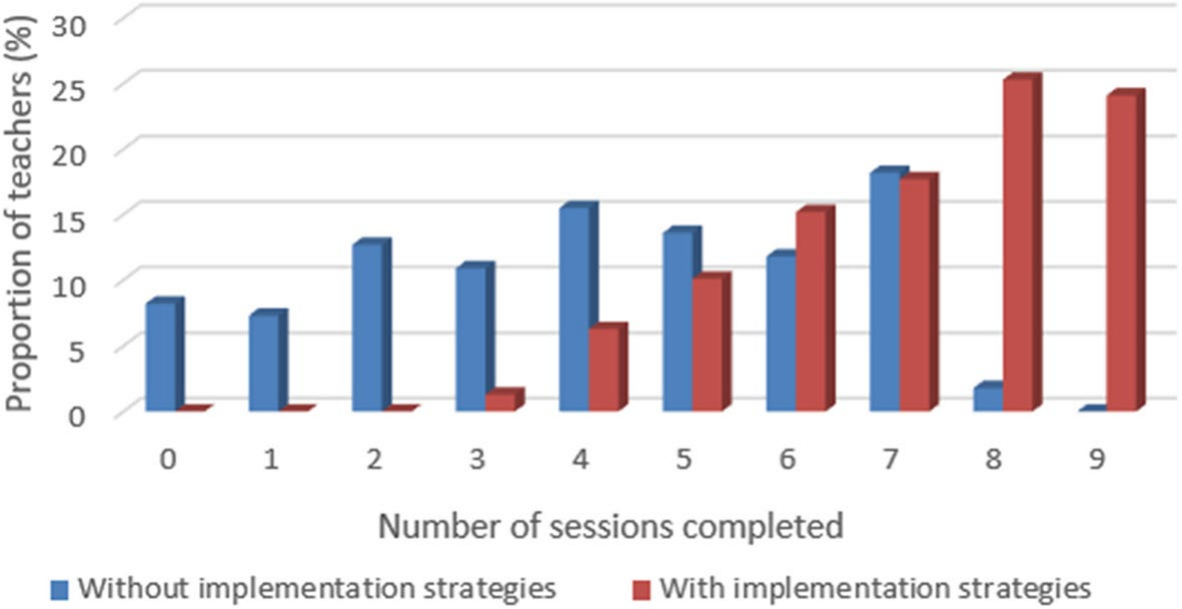
Number of sessions in FOYC+ClmPACT taught by teachers: usual vs. enhanced implementation

**Fig. 2:**
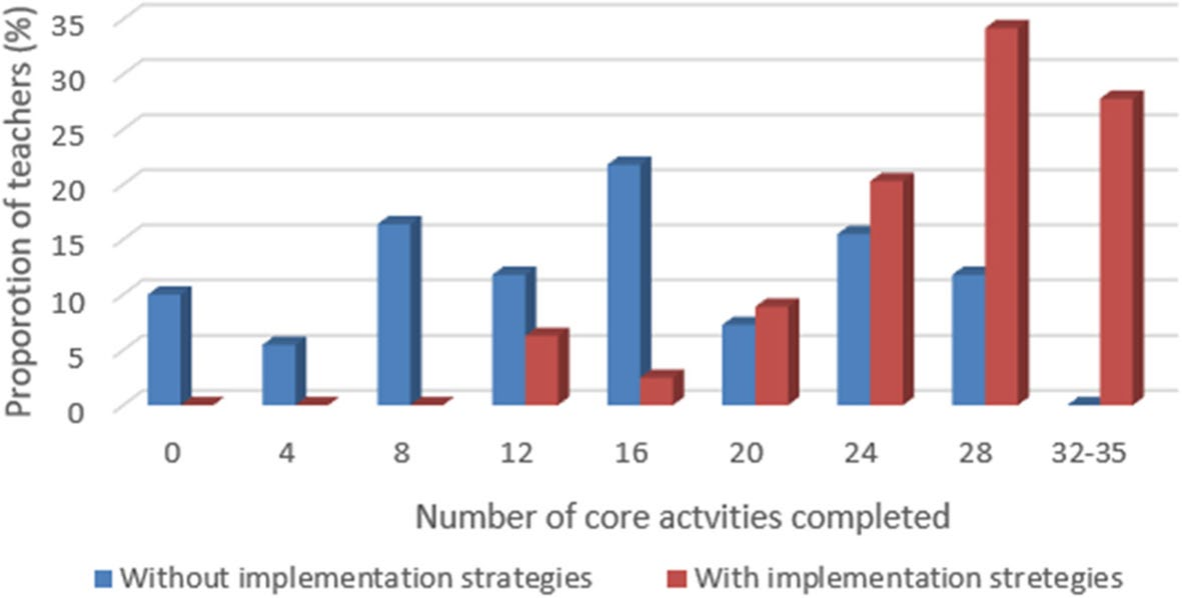
Number of core activities in FOYC+ClmPACT taught by teachers: usual vs. enhanced implementation

In the 2011–2012 trial (standard implementation), the 110 teachers who participated in the first wave of national implementation of the program in New Providence taught 16.3 core activities and 4.4 sessions on average (Figs. 1 and 2). Two-thirds of the teachers attended the training workshops, but they did not receive any other technical support (such as implementation monitoring and mentoring). In 2019–2020, 79 teachers participated in the first wave of the ongoing national implementation of the program in New Providence where newly developed implementation monitoring and assistance strategies are deployed. The teachers taught 28.3 out of 35 core activities (28.3 vs. 16.3, t = 10.80, *p* < 0.001) and 7.2 out of 9 sessions in FOYC+CImPACT (7.2 vs. 4.4, t = 9.14, *p* < 0.001) (Figs. 1 and 2). Approximately 82% of the teachers taught > 24 core activities during enhanced implementation in 2019–2020. By contrast, only 27% taught > 24 core activities in 2011–2012 (Fig. 2). Teachers taught > 80% of the intervention curriculum in 2019–2020 despite the massive interruption caused by Hurricane Dorian and COVID-19.

### Association between teachers/schools’ factors and teacher’s implementation fidelity in 2019–2020

As shown in Table 1, number of years of teaching experience, teacher’s education, attendance of the FOYC training workshop, having other priorities, and previous experience of teaching an HIV risk reduction program were not associated with the implementation of FOYC+CImPACT. Teachers who had a “very good” or “excellent” school coordinator in their schools taught more core activities than those who had a “satisfactory” school coordinator (30.4 vs. 29.6 vs. 22.3, F = 18.54, *p* < 0.001). Teachers who worked in a school which had a “very good” mentor, taught more core activities than those teachers who did not have a mentor or had only a “satisfactory” mentor (30.4 vs. 27.6; t = 2.96; *p* = 0.004). Teachers’ perception of the importance of the intervention and teachers’ sense of ownership of the FOYC curriculum (e.g., as a “Bahamian intervention”) were not associated with implementation (Table 1).

**Table 1:** Association between teacher’s characteristics, training experience, performance of their school coordinators and mentors and number of core activities and sessions taught in the classroom among 79 grade six school teachers in 2019–2020

Variables	n	Number of core activities completed (0–35)	Number of core activities taught exactly as outlined in the manual (0–35)	Number of sessions taught (0–9)
Total years as teacher or guidance counselor				
1 ~ 5 years	9	28.6(4.6)	25.1(4.0)	6.9(2.0)
6 ~ 10 years	18	29.7(4.9)	23.9(6.4)	7.5(1.4)
> 10 years	50	27.5(5.5)	21.8(7.4)	7.0(1.6)
F test		1.12	1.25	0.73
Education level				
Associate degree/teaching certificate	4	26.5(1.7)	19.3(8.7)	6.5(1.0)
Bachelor degree	60	28.1(5.7)	23.1(7.0)	7.0(1.6)
Master degree	12	28.8(4.4)	22.5(5.6)	7.9(1.6)
F test		0.28	0.60	1.97
Attended a FOYC training workshop				
Yes	64	28.1(5.6)	22.4(7.2)	7.0(1.7)
No	15	29.3(4.0)	24.4(5.1)	7.9(1.1)
t test		−0.79	−1.02	− 1.95
Prior experience of teaching HIV risk reduction intervention				
Yes	12	29.6(3.0)	23.9(7.0)	7.3(1.5)
No	65	28.0(5.7)	22.4(6.9)	7.1(1.6)
t test		1.43	0.69	0.37
Having other teaching priorities				
Yes	27	28.3(6.1)	22.6(6.5)	6.9(1.8)
No	44	28.5(4.9)	23.3(7.1)	7.3(1.5)
t test		−0.22	−0.41	−1.00
Importance of FOYC for the grade six students in your school				
Very important	72	28.1(5.4)	22.3(6.9)	7.1(1.6)
Somewhat important	5	29.8(4.8)	26.8(4.4)	7.2(2.2)
t test		−0.70	−1.43	−0.16
FOYC is a Bahamian curriculum				
Very much so	49	27.6(5.7)	22.4(7.4)	6.9(1.6)
Somewhat	26	28.9(4.6)	22.3(5.7)	7.5(1.4)
t test		−1.04	0.07	− 1.74
Performance of school coordinators				
Satisfactory	16	22.3(6.4)	18.5(8.1)	6.4(1.7)
Very good	47	29.6(3.7)	24.4(6.2)	7.1(1.6)
Excellent	16	30.4(4.1)	22.2(5.8)	7.9(1.1)
F test		18.54***	5.01**	3.77*
Performance of mentors				
No mentors/satisfactory	59	27.6(5.8)	22.3(7.1)	6.9(1.6)
Very good	20	30.4(2.6)	24.3(5.8)	7.8(1.6)
T test		−2.96^**^	−1.16	−2.01^*^

*Note*: * *P* < 0.05; ** *P* < 0.01; *** *P* < 0.001

The results of the mixed-effects model indicate that performance of the school coordinators at each school was significantly related to teachers’ implementation fidelity (Table 2). Compared to teachers who had a “satisfactory” school coordinator, teachers who had an “excellent” or “very good” school coordinator in their schools taught more core activities after controlling for education, comfort level, confidence, attitudes towards sex education, perceived principal support, self-efficacy and clustering effects of school. (β = 6.83, t = 2.40, *p* = 0.02; β = 5.94, t = 2.67, *p* = 0.01). Teachers’ self-efficacy was predictive of implementation fidelity (β = 2.08; t = 2.67, *p* = 0.01). School random effects were significant (β = 14.52, t = 2.32, *p* = 0.01), indicating significant variation among schools with regard to teachers’ implementation fidelity (number of core activities taught).

**Table 2:** Mixed-effects model assessing the association between implementation strategies and teachers’ implementation fidelity

*Fixed effect*	*β*	*SE*	*t*	*p*
Intercept	16.535	5.463	3.03	0.007
Teachers’ education				
Associate degree/teaching certificate	−1.375	2.107	−0.65	0.517
Bachelor’s degree	−0.532	1.106	−0.48	0.633
Master degree (ref)	0			
Comfort level with the curriculum	1.856	1.279	1.45	0.154
Confidence in implementing core activities	−0.167	0.616	−0.27	0.787
Attitudes towards sex education in schools	−1.942	0.995	−1.95	0.058
Perceived principal support	0.637	0.728	0.88	0.386
Teachers’ self-efficacy	2.081	0.780	2.67	0.011
Performance of school coordinators				
Excellent	6.829	2.846	2.40	0.021
Very good	5.943	2.223	2.67	0.011
Satisfactory	0			
Performance of site-based mentors				
Very good	2.229	2.390	0.93	0.357
Satisfactory	−0.564	2.193	−0.26	0.798
No mentor	0			
*Random effect*				
School	14.519	6.258	2.32	0.010

*Note*: * *P* < 0.05; ** *P* < 0.01; *** *P* < 0.001

### Association between teacher’s implementation degree and student outcome in 2019–2020

Baseline grade 6 student survey were conducted by the research team in the classroom in fall of 2019. Due to the COVID-19 pandemic and school closure, administration of the classroom student follow-up survey was changed to an online format and was delayed until the beginning of grade 7. The mean age of the students was 10.5 years (range 9–15 years) at baseline; 50.7% of the students were female. At baseline, 2252 students completed program evaluation assessments; 850 students completed end of the year assessments. Overall, students’ HIV/AIDS knowledge, reproductive health skills, self-efficacy, and intention to use protection were significantly higher at the end of the year than at the beginning of the school year (knowledge 11.2 vs. 8.8, t = 19.67, *p* < 0.001; skills 4.6 vs. 3.8, t = 13.81, *p* < 0.001; self-efficacy 2.8 vs. 2.5, t = 8.01, *p* < 0.001; and intention 3.9 vs. 2.9, t = 14.89, *p* < 0.001). The results of the mixed-effects models indicate that the degree of implementation (number of sessions completed) was significantly associated with increased reproductive health skills, self-efficacy, and intention to use condom protection at follow-up (AIDS knowledge was marginally significant). Baseline scores of AIDS knowledge, self-efficacy, and intention to use condom protection were predictive of respective outcome at follow-up. School random effects were not significant in the intention models (Table 3).

**Table 3:** Mixed-effects models assessing the impact of teachers’ implementation degree on students’ outcomes

Variables	Estimated models											
	HIV/AIDS knowledge			Preventive reproductive health skills			Self-efficacy			Intention to use protection		
	β	SE	*t*	β	SE	*t*	β	SE	*t*	β	SE	*t*
*Fixed effect*												
Intercept	3.984	5.846	0.68	3.018	2.042	1.48	1.064	1.661	0.64	2.444	2.373	1.03
Age	0.286	0.542	0.53	− 0.009	0.172	− 0.05	0.251	0.136	1.58	− 0.072	0.195	−0.37
Gender												
Male	0.124	0.286	0.43	0.146	0.153	0.95	0.065	0.091	0.72	0.087	0.185	0.47
Female (ref)												
Baseline student outcome	0.254	0.105	2.43^*^	0.129	0.117	1.10	0.325	0.178	1.82^#^	0.444	0.122	3.63^***^
Implementation degree (number of sessions completed)	0.889	0.516	1.72^#^	0.523	0.258	2.03^*^	0.221	0.097	2.27^*^	0.118	0.054	2.18^*^
*Random effect*												
School^a^	0.124	0.195	0.63	0.103	0.097	1.06	0.040	0.034	1.20	0.193	0.127	1.51^#^

*Note*: # *P* < 0.10; * *P* < 0.05; ** *P* < 0.01; *** *P* < 0.001. ^a^ z test

### Relationships among factors influencing teachers’ implementation fidelity

The strength of associations between factors influencing teachers’ self-efficacy and implementation was examined using Pearson correlation coefficients (Table 4). Teachers’ comfort level with the curriculum, confidence in teaching five core activities, attitudes towards sex education, and perceived principal support were significantly related to increased self-efficacy (r = 0.31–0.54, *p* < 0.01). Perceived principal support and performance of school coordinators were significantly related to teachers’ degree of implementation (r = 0.23–0.43, *p* < 0.05). Teachers’ comfort level was significantly related with teachers’ confidence (r = 0.54, *p* < 0.001) and positive perceptions of sex education in schools (r = 0.41, *p* < 0.001). Teachers’ confidence was also related to their perceptions of sex education in schools (r = 0.32, *p* < 0.01).

**Table 4:** Bivariate correlation among factors influencing teachers’ self-efficacy and implementation

Variables	1	2	3	4	5	6	7	8	9	Mean	SD
1. Comfort level with the curriculum	1.00									2.50	0.49
2. Confidence in implementing core activities	0.54^c^	1.00								4.20	0.92
3. Attitudes towards sex education in schools	0.41^c^	0.32^b^	1.00							3.73	0.54
4. Perceived principal support	0.17	0.07	0.13	1.00						3.75	0.69
5. Self-efficacy	0.54^c^	0.31^b^	0.36^b^	0.36^b^	1.00					3.67	0.74
6. Performance of school coordinators	−0.17	−0.04	−0.19	−0.04	− 0.11	1.00				2.00	0.64
7. Performance of mentors	−0.16	0.11	0.10	− 0.29^b^	−0.07	0.23^a^	1.00			1.75	0.84
8. Number of core activities taught	−0.06	−0.05	− 0.12	0.23^a^	0.02	0.43^c^	0.21	1.00		28.29	5.32
9. Number of core activities taught exactly as outlined in the manual	0.14	−0.01	0.07	0.31^b^	0.23^a^	0.11	0.13	0.71^c^	1.00	22.77	6.85

*Note*: ^a^ *p* < 0.05; ^b^ *p* < 0.01; ^c^ *p* < 0.001. SD = Standard deviation. Score range:1 ~ 5 for confidence, sex education, principal support and self-efficacy

In addition, performance of school coordinators was related performance of mentors in that school (r = 0.23, *p* < 0.05).

**Fig. 3:**
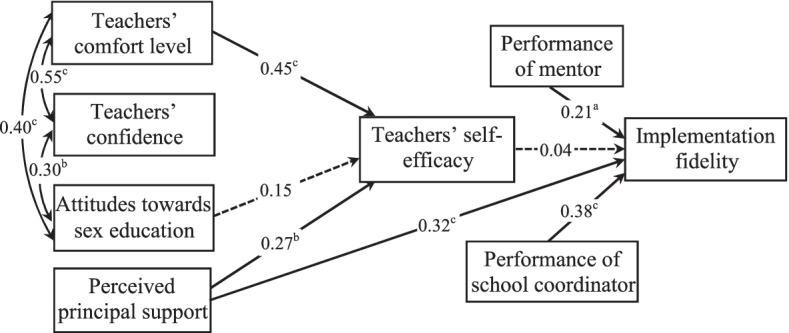
Revised structural model showing relationships among factors influencing teachers’ implementation fidelity. Standardized path coefficients are shown. Note: a *p* < 0.05; b *p* < 0.01; c *p* < 0.001

Structural equation modeling demonstrated relationships among factors and their direct and indirect effect on implementation fidelity (i.e., number of core activities taught) (Fig. 3). There were six manifest exogenous variables (teachers’ comfort level, teachers’ confidence, attitudes towards sex education, perceived principal support, performance of school coordinator, and performance of mentor) and two manifest endogenous variables (self-efficacy, implementation fidelity) in the model. In modifying the initial model, we removed the paths from teachers’ comfort level, teachers’ confidence, and attitudes towards sex education to implementation fidelity, and the path from teachers’ confidence to self-efficacy as they were nonsignificant.

In the revised model, teachers’ comfort level and perceived principal support predicted increased self-efficacy. Performance of school coordinator, performance of mentor, and perceived principal support had positive direct effects on implementation fidelity. The direct effect of self-efficacy on implementation fidelity was not significant. Teachers’ confidence and attitudes towards sex education were positively related to teacher’s comfort level. The overall fit of the revised path model was excellent (CFI = 0.988, TLI = 0.983, RMSEA = 0.03, chi-square/df = 1.08; *p* = 0.37; SRMR = 0.07). The analysis revealed an R^2^ value of 0.35 for teachers’ self-efficacy and of 0.32 for implementation fidelity.

## Discussion

This study investigated the implementation of an evidence-based intervention in collaboration with The Bahamas Ministries of Education and Health by comparing data from standard and enhanced implementation trials, which allows assessment of the relative contribution of an enhanced implementation approach to both implementation fidelity and program outcomes. The findings reveal that teachers taught 80% of the intervention curriculum on average with newly developed implementation strategies compared to 50% curriculum delivery in usual implementation (where only teacher training was provided), indicating a 60% increase from the average level of school-based implementation of prevention programs [16, 37]. This study demonstrated that school-based HIV prevention intervention can be implemented with a high degree of fidelity when effective implementation strategies (implementation monitoring and site-based assistance and mentorship) are in place.

Teachers’ comfort levels with the curriculum, their confidence in teaching core activities were significantly related to increased self-efficacy, which in turn was positively related to teachers’ degree of implementation. Teachers’ characteristics such as teacher’s comfort level, confidence, and self-efficacy in teaching the curriculum are the “inner context” factors in EPIS model that play crucial roles in the implementation phase. These are potentially modifiable factors related to program delivery [10]. Therefore, teacher training efforts should be directed towards enhancing teachers’ competency regarding teaching the intervention curriculum (especially sensitive topics such as condom use demonstration). Interestingly, self-efficacy is significantly related to number of core activities taught exactly as outlined in the manual but is not related to total number of core activities taught. Inversely, the performance of school coordinators is significantly related to the total number of core activities taught, but it is not related to the number of core activities taught exactly as outlined in the manual. These results may imply that teachers’ degree of implementation is highly impacted by their school coordinators but the quality of intervention delivery is mainly determined by teachers’ self-efficacy in teaching the FOYC+CImPACT intervention. Consistent with previous research [37], our study indicate that teachers’ degree of implementation is significantly associated with improved student outcomes (including reproductive health skills, self-efficacy, and intention to use condom protection).

Although the MOE fully supported the implementation of the FOYC + CImPACT intervention among grade six students, teacher’s perception of school administrators’ support varied from school to school. Organizational factor such as perceived principal support (“inner context” factors) is significantly related to teachers’ self- efficacy and implementation fidelity, which underscores the importance of school-level support to the success of school-based implementation. Our finding that teacher’s perception of the importance of the intervention was not related to implementation fidelity is not consistent with prior studies [21]. This inconsistency may be because a very small portion (< 6%) of the teachers did not perceive the importance of HIV prevention or FOYC+CImPACT intervention for Grade 6 youth. In enhanced implementation, school coordinators (and mentors) demonstrated great impact on teachers’ implementation. As many of the school coordinators for our implementation study are also vice principals of the schools, teachers might feel more obligated to implement the intervention curriculum despite their training, teaching experience and perception of the importance of HIV or FOYC+CImPACT.

In contrast to the performance of mentors, the performance of school coordinator was strongly associated with teachers’ implementation which has become a pivotal strategy in our implementation. This result points to the importance of biweekly implementation monitoring to enhance program implementation, which is consistent with prior research and EPIS model [28, 39]. It should be noted that half of the schools in this study did not have a mentor to support the teachers who were at risk for not implementing the intervention curriculum and 25% of the schools only had a satisfactory mentor due to the interruption of the COVID-19 (e.g., the periodical school closures would have impeded sited-based mentoring). Our future data will further examine the impact of mentor (and the number of mentoring sessions) on implementation fidelity. Performance of the mentors was significantly related to implementation fidelity in bivariate analysis but this relationship became non-significant in multivariate analysis due to the correlational nature of the two variables.

Our study is a nationwide implementation trial of an evidence-based HIV prevention intervention in a relatively understudied Caribbean cultural context with a longitudinal study design and a large sample size. However, there are several potential limitations in this study. First, our findings were based on teachers’ and students’ self-reports, which are subject to social desirability and recall bias. It is possible that teachers over-reported their level of implementation. In the current study, trained observers independently observed and assessed approximately 10% of each teacher’s classes. We found that the observer-teacher agreement was high (about 90%), indicating that teachers’ self-reports of their implementation are reliable. Second, the student follow-up rate is low (38%). This is because the baseline student data were collected in classrooms in September and October 2019. The follow-up student data were collected online due to the school closure in April 2020 caused by the COVID-19. We were only able to follow 38% of the students in fall 2020 at the time these students entered their Grade 7. The follow-up student survey was originally scheduled in May–June 2020. The other reasons for attrition included students’ non-identification of their grade 6 teachers in the delayed follow-up survey and loss of contact due to students’ graduation from primary school and transferring into non-government middle schools (private or religious-based schools). Our student outcome evaluation is affected by relatively low student follow-up rate. Third, we only identified and trained 12 high-performing teachers (mentors) to assist at-risk and moderate-performing teachers due to the interruption of the COVID-19. Half of the schools in this study did not have a mentor. Our team will identify and train more mentors to provide site-based assistance and mentoring in the subsequent years.

## Conclusion

Our study demonstrated that an evidence-based HIV prevention intervention can achieve a high degree of implementation when delivered with enhanced implementation strategies and implementation monitoring. Our study highlights the importance of school-level support in promoting teachers’ implementation. Future program implementers should consider the purposeful selection and training of school coordinators and mentors to support low-implementing teachers as a potentially important strategy when attempting to achieve high-quality implementation of school-based interventions. Our findings address recognized gaps in knowledge regarding effective implementation strategies and teacher implementation support in large scale implementation of effective prevention programs in schools.

## Data Availability

The datasets analyzed during the current study available from the corresponding author on reasonable request.
